# Profiling Culturally Responsive Care: Intercultural Communication and Empathy in the Nursing Workforce

**DOI:** 10.3390/healthcare14081095

**Published:** 2026-04-20

**Authors:** Fatma Ayşin Kurak, Ersin Taşatan, Hayriye Deniz Şelimen

**Affiliations:** 1 Department of Nursing, Cyprus Aydın University, Turkish Republic of Northern Cyprus, Kyrenia 99320, Türkiye; 2 Department of Orthopedics and Traumatology, Prof. Dr. Cemil Tascioglu City Hospital, Istanbul 34384, Türkiye; 3 Department of Nursing, Final University, Turkish Republic of Northern Cyprus, Kyrenia 99300, Türkiye

**Keywords:** intercultural communication competence, empathy, nursing, culturally responsive care, perspective taking

## Abstract

**Highlights:**

**What are the main findings?**

- Nurses reported above-midpoint intercultural communication competence (ICC) and empathy, with cognitive ICC exceeding affective ICC.
- ICC and empathy were moderately associated (ICC total–empathy total: r = 0.607), and intrinsic professional choice (willingly choosing nursing) was linked to higher scores across domains.

**What are the implications of the main findings?**

- Nursing education and in-service programs should prioritize experiential and reflective training to strengthen affective ICC and perspective taking.
- Workforce strategies should support intrinsic motivation and career-long development, as competencies varied by age/experience more than by hospital sector.

**Abstract:**

**Background/Objectives:** Culturally responsive care requires both intercultural communication competence (ICC) and empathy; however, these constructs are often examined separately in nursing research. This study aimed to (i) describe nurses’ ICC and empathy levels, (ii) test the association between ICC and empathy, and (iii) examine group differences by selected demographic and professional variables. **Methods:** A quantitative, cross-sectional correlational design was conducted with 300 nurses recruited from state and private hospitals. ICC was measured using the Arasaratnam Intercultural Communication Competence Scale (cognitive, affective, and total), and empathy was assessed using the 18-item Jefferson Scale of Empathy (compassionate care, perspective taking, standing in the patient’s shoes, and total). Data were analyzed with descriptive statistics, Pearson correlations, independent-samples t-tests, and one-way ANOVAs with Scheffé post hoc tests (α = 0.05). **Results:** Both ICC and empathy were above the scale midpoint. Cognitive ICC (M = 4.71, SD = 1.42) exceeded affective ICC (M = 4.35, SD = 1.34), and total empathy was high (M = 4.50, SD = 0.90), with compassionate care as the highest subscale (M = 4.60, SD = 1.10). ICC total was moderately correlated with total empathy (r = 0.607, *p* < 0.05); affective ICC correlated with compassionate care (r = 0.455) and perspective taking (r = 0.493). Male nurses reported higher ICC than female nurses (*p* < 0.05), while empathy did not differ by gender. Younger nurses (20–29) scored higher in ICC and empathy than older groups, and nurses with ≥28 years of experience also showed elevated levels. Nurses who willingly chose nursing had higher ICC and empathy across dimensions (all *p* < 0.001). Hospital type showed minimal differences except for “standing in the patient’s shoes” (private > state, *p* = 0.04). **Conclusions:** ICC and empathy were generally high and interrelated among nurses, with meaningful variation across workforce characteristics. Training should emphasize experiential and reflective approaches to strengthen affective ICC and perspective taking, while organizational strategies should foster intrinsic motivation and support professional development across career stages.

## 1. Introduction

Culture, as a dynamic system of values, beliefs, norms, and practices, provides the framework through which individuals and societies interpret their environment and interact with one another. It encompasses both tangible (e.g., art, technology) and intangible (e.g., language, rituals, symbols) components that shape social identity and collective memory. Cultural identity, defined as an individual’s sense of belonging to a particular group and the internalization of its values, plays a crucial role in guiding behavior and social interaction [1,2,3,4,5]. In an era of globalization and migration, multiculturalism has emerged as a paradigm that emphasizes tolerance, inclusivity, and the coexistence of multiple cultural identities within the same social environment [6,7]. These dynamics highlight that culture is not static but continually shaped by historical, economic, and technological developments, with education and health care serving as key domains where cultural diversity is most visible.

Intercultural communication (ICC) emerges as a pivotal concept in contexts where individuals from different cultural backgrounds interact. Defined as the process through which people negotiate meaning across cultural boundaries, ICC extends beyond linguistic competence to include sensitivity to cultural norms, values, and relational expectations [8,9]. Theories such as face-negotiation, social identity, and communication accommodation illuminate how cultural differences influence communicative strategies, power dynamics, and the management of uncertainty in interpersonal encounters. In nursing, ICC is indispensable, as it directly impacts the therapeutic relationship, the accuracy of patient assessments, and the effectiveness of care delivery [10,11,12,13]. By facilitating trust and mutual understanding, intercultural communication enables nurses to address the needs of increasingly diverse patient populations, while mitigating the risk of miscommunication, stereotyping, or inequitable care.

Empathy, defined as the ability to perceive, understand, and respond to the emotions and perspectives of others, complements ICC by addressing the affective and relational dimensions of care [14,15]. Historically rooted in the philosophical notion of Einfühlung, empathy has evolved into a multidimensional construct encompassing emotional, cognitive, and compassionate components. Within nursing, empathy is recognized as a cornerstone of professional practice, enhancing patient satisfaction, adherence to treatment, and psychological well-being [16]. It also fosters collaboration within healthcare teams, supports ethical decision-making, and protects against professional burnout. Given the increasing cultural heterogeneity in healthcare settings, the integration of ICC and empathy represents not only a clinical necessity but also a moral imperative, ensuring that care remains equitable, patient-centered, and culturally responsive.

In contemporary healthcare environments, nurses are frequently required to provide care to patients from diverse cultural, social, and linguistic backgrounds. In this context, intercultural communication competence (ICC) and empathy are not merely desirable skills, but essential components of safe, effective, and patient-centered care. ICC enables nurses to understand, respect, and effectively interact with individuals from different cultural groups, while empathy supports the emotional and relational aspects of care, allowing for trust, rapport, and holistic patient support. Despite their recognized importance, these two constructs are often examined in isolation in the literature. By exploring them simultaneously, this study contributes to a deeper understanding of how cognitive, affective, and relational dimensions of intercultural communication are intertwined with the empathic capacity that underpins compassionate nursing practice.

Beyond documenting average levels, this study further examines how ICC and empathy differ across demographic and professional characteristics. This approach is particularly significant, as it identifies both modifiable factors (e.g., willingly choosing the nursing profession, working with enthusiasm) and structural factors (e.g., years of professional experience, hospital type) that influence communication and empathic abilities. The findings, based on a robust sample of 300 nurses, provide empirical benchmarks for workforce development, training curricula, and policy frameworks aimed at improving the quality of nursing care. Ultimately, the study highlights the necessity of integrating ICC and empathy into nursing education, in-service training, and institutional quality improvement initiatives, thereby promoting more equitable and culturally responsive healthcare delivery.

## 2. Materials and Methods

### 2.1. Study Design

This study employed a quantitative, cross-sectional correlational design. It aimed to examine nurses’ levels of intercultural communication competence and empathy, to determine the relationship between these variables, and to evaluate whether they differ according to selected demographic and occupational characteristics (gender, age, marital status, total years of employment, choosing the profession willingly, working with love/commitment, hospital type, and work shift pattern).

### 2.2. Population and Sample

The study population consisted of all nurses working in the Turkish Republic of Northern Cyprus (TRNC). According to official records, there were 1285 nurses in total, including 940 working in public hospitals (73.2%) and 345 in private hospitals (26.8%).

The minimum required sample size was calculated using the finite population correction formula. Assuming a 95% confidence level, a 5% margin of error, and the most conservative population proportion (*p* = 0.50), the minimum required sample size was determined as n = 296 (Krejcie & Morgan, 1970) [17]. The use of *p* = 0.50 provides the maximum variance assumption and therefore yields the most conservative estimate of the required sample size when the exact population proportion is unknown. This sample size was considered sufficient for the planned inferential analyses, including group comparisons using independent-samples *t*-tests and one-way ANOVA.

To ensure representativeness, stratified random sampling was applied based on hospital type. Participants were proportionally allocated to strata according to the population distribution, and within each stratum nurses were selected using simple random sampling. A total of 313 questionnaires were collected. This number exceeded the minimum required sample size and ensured adequate representation of both strata. Thirteen questionnaires (4.15%) were excluded due to incomplete responses on primary study variables (ICC and/or empathy scale items). No cases were excluded as outliers after screening.

The final analytic sample comprised 300 nurses, exceeding the minimum required sample size and closely reflecting the population distribution by hospital type. All retained questionnaires were complete for the primary outcome variables (ICC and JSE total and subscale scores). Questionnaires excluded from analysis contained missing responses on key scale items and were therefore omitted from further analysis. Because the final dataset consisted of complete cases for all variables included in inferential analyses, correlations and group comparisons (*t*-tests and ANOVA) were conducted on n = 300 using complete-case (listwise) analysis.

Although population-level data were not available for other variables, the distributions of gender, age, and years of professional experience were consistent with the known structure of the nursing workforce. Therefore, sampling imbalance is unlikely to have biased the results or affected the validity of subsequent analyses.

### 2.3. Data Collection Instruments

Demographic Information Form: A structured demographic form was developed based on the literature and expert opinion. The form included items on gender, age, marital status, total years of employment, hospital type, work shift pattern, choosing the profession willingly, and working with love/commitment (Table 1).

Intercultural Communication Competence Scale: Intercultural communication competence was assessed using the Intercultural Communication Competence Scale developed by Arasaratnam [18]. The scale consists of 10 items rated on a 7-point Likert scale, with five reverse-coded items. Higher scores indicate greater intercultural communication competence.

The Turkish adaptation and validation were conducted by Sürücü et al. [19] following recommended translation procedures. In the present study, internal consistency was acceptable, with a Cronbach’s alpha of 0.756 for the total scale, 0.722 for the cognitive subscale, and 0.747 for the affective subscale.

Jefferson Scale of Empathy: Empathy was measured using the Jefferson Scale of Empathy, developed by Hojat and Herman. The scale is a 7-point Likert-type self-report instrument designed to assess empathy in healthcare settings. The Turkish validity and reliability study was performed by Yanık and Saygılı [20].

Construct validity was supported by KMO = 0.78 and a significant Bartlett’s test (*p* < 0.001). In this study, the scale demonstrated good reliability (Cronbach’s alpha = 0.798 for the total scale). Subscale alpha coefficients were 0.727 (Compassionate Care), 0.781 (Perspective Taking), and 0.754 (Standing in the Patient’s Shoes).

### 2.4. Data Collection Procedure

Data were collected via a three-part self-report questionnaire administered on site. Responses were screened for completeness and outliers. Normality was evaluated using skewness and kurtosis (|±1.5| threshold), supporting the use of parametric tests. Reverse-keyed ICC items were recoded prior to computing subscale and total scores. A total of 313 questionnaires were returned; 13 (4.15%) were excluded due to incomplete responses on primary scale items (ICC and/or JSE). The final analytic dataset (n = 300) contained complete responses for all study variables used in inferential analyses; therefore, all correlations and group comparisons were conducted on n = 300 using complete-case analysis.

### 2.5. Statistical Analysis

Statistical analyses were performed in SPSS v24, with psychometric checks cross-validated in AMOS v18. For RQ1 and RQ2, descriptive statistics (means and standard deviations) were calculated for intercultural communication competence (ICC) cognitive, affective, and total scores, as well as for the empathy subscales (compassionate care, perspective taking, standing in the patient’s shoes) and the total empathy score; internal consistency (Cronbach’s α) was reported for all scales and subscales. For RQ3, Pearson correlation coefficients were computed among ICC dimensions and empathy dimensions and summarized as a correlation matrix with corresponding *r* and *p* values. For RQ4, group differences were examined using independent-samples *t*-tests for gender, willingly choosing the profession, and hospital type, and one-way ANOVA across age bands and total years of experience with Scheffé post hoc tests when the omnibus *F* was significant. Assumptions were checked via Levene’s test for homogeneity of variances; Scheffé was applied when assumptions were met, and robust alternatives were considered and reported when violated. A two-tailed significance threshold of α = 0.05 was adopted for all analyses, and effect sizes (Cohen’s *d* for *t*-tests; η^2^ for ANOVA) were calculated to support interpretation.

### 2.6. Ethical Considerations

The study was conducted in accordance with ethical principles for research involving human participants, including voluntary participation, informed consent, and confidentiality. Ethical approval was obtained from the Cyprus Aydın University Ethics Committee (approval number 2024/07.004, approved on 30 July 2024).

### 2.7. Data Availability and AI Disclosure

The datasets generated during the current study are available from the corresponding author upon reasonable request. Generative AI tools were not used for data generation, analysis, or interpretation.

## 3. Results

The analysis revealed that nurses reported intercultural communication competence levels above the scale midpoint, suggesting that the majority of participants perceive themselves as adequately prepared to interact with individuals from diverse cultural backgrounds. Specifically, the mean score for the cognitive dimension was M = 4.71 (SD = 1.42), while the affective dimension averaged M = 4.35 (SD = 1.34). The higher scores on the cognitive domain indicate that nurses are relatively stronger in their knowledge, awareness, and conceptual understanding of cultural diversity compared to their affective readiness to engage with patients in emotionally complex intercultural contexts. The relatively large standard deviations, particularly in the cognitive dimension, highlight considerable variability across respondents, suggesting that while some nurses demonstrate advanced intercultural awareness, others may require targeted training and support to strengthen these skills. Overall, the results demonstrate that nurses’ intercultural communication competence is at an above-average level but unevenly distributed across cognitive and affective subcomponents (Table 2).

Overall empathy levels among nurses were also above average, with a total mean score of M = 4.50 (SD = 0.90), indicating that participants generally possess a well-developed ability to understand and respond to patients’ emotional needs. Examination of subdimensions revealed that compassionate care had the highest mean score (M = 4.60, SD = 1.10), underscoring nurses’ strong inclination to provide care characterized by sensitivity and emotional support. The subdimension standing in the patient’s shoes averaged M = 4.45 (SD = 1.42), suggesting that many nurses can successfully adopt the perspective of their patients, although the relatively high standard deviation indicates heterogeneity in this ability. The perspective taking subdimension was similar in level (M = 4.44, SD = 0.99), reflecting a consistent though slightly less pronounced capacity to consider patients’ viewpoints. Taken together, these findings point to a generally high level of empathic engagement among nurses, with particular strengths in compassionate caregiving behaviors.

Correlation analyses showed statistically significant and positive relationships between ICC dimensions and empathy dimensions, indicating that the two constructs are interdependent and mutually reinforcing. The total ICC score demonstrated a moderate positive correlation with total empathy (r = 0.607, *p* < 0.05), suggesting that as nurses’ intercultural competence increases, so does their empathic responsiveness. Among ICC dimensions, the affective component showed a particularly strong association with overall ICC (r = 0.860), confirming its central role in shaping communication competence. Furthermore, affective ICC was moderately correlated with compassionate care (r = 0.455) and perspective taking (r = 0.493), indicating that emotional readiness to engage with cultural differences translates into enhanced empathy. On the empathy side, perspective taking and total empathy were very strongly correlated (r = 0.911), underscoring that the ability to adopt patients’ viewpoints is a core driver of overall empathy. Collectively, these results highlight the close linkage between intercultural communication skills and empathic caregiving, with improvements in one domain likely contributing to gains in the other (Table 3).

Independent-samples *t*-tests revealed significant gender differences in ICC. Male nurses scored higher on both the cognitive (M = 5.01 vs. M = 4.57, t = 2.497, *p* = 0.013) and affective (M = 4.69 vs. M = 4.19, t = 2.497, *p* =0.002) dimensions compared to female nurses. This suggests that men may report greater confidence in both their knowledge of cultural diversity and their affective engagement in intercultural encounters. However, empathy scores did not differ significantly by gender across any of the subscales, indicating that empathic tendencies are relatively stable and not influenced by gender in this sample (Table 4).

Significant age-related differences emerged across ICC and empathy. Younger nurses, particularly those aged 20–29, scored the highest in both cognitive ICC (*F* = 13.643, *p* < 0.001) and affective ICC (*F* = 8.209, *p* < 0.001). Post hoc analyses showed that this youngest group significantly outperformed older nurses, while those aged 30–39 and 40–49 also reported higher ICC than the 50+ group. For empathy, compassionate care (*F* = 7.140, *p* < 0.001) and perspective taking (*F* = 13.153, *p* < 0.001) were also highest in the 20–29 group, declining progressively with age. By contrast, standing in the patient’s shoes did not differ significantly by age group (*F* = 2.093, *p* = 0.101). These results suggest that younger nurses may be more adaptable and sensitive to intercultural and empathic demands, whereas older nurses may require ongoing professional development in these areas (Table 5).

Experience levels were significantly associated with ICC and empathy. Nurses with ≥28 years of experience scored highest on ICC cognitive (*F* = 3.464, *p* = 0.017) and ICC affective (*F* = 8.047, *p* < 0.001), as well as on empathy subscales including compassionate care (*F* = 8.660, *p* < 0.001) and perspective taking (*F* = 7.786, *p* < 0.001). This pattern suggests that prolonged exposure to diverse patient populations may enhance both intercultural communication and empathy. Although standing in the patient’s shoes also yielded a significant omnibus effect (*F* = 3.594, *p* = 0.014), post hoc tests did not confirm significant pairwise differences, indicating that the effect may be more subtle. Therefore, this finding should be interpreted with caution, as the absence of significant pairwise differences suggests that the observed effect may be modest rather than reflecting a clear progression across experience levels. Overall, the findings support the idea that clinical experience contributes to strengthening communication and empathy, though certain dimensions may plateau after many years of practice (Table 6).

Strong differences were observed between nurses who reported choosing the profession willingly and those who did not. Those who willingly entered nursing scored significantly higher on ICC cognitive (t = 4.482, *p* < 0.001) and ICC affective (t = 5.053, *p* < 0.001), as well as on all empathy subscales (all *p* < 0.001). These results suggest that intrinsic motivation for entering the profession is a powerful determinant of both intercultural communication and empathy. In other words, nurses who actively chose nursing are more likely to invest in both the cognitive and emotional aspects of communication and to demonstrate compassion, perspective taking, and role reversal in patient interactions (Table 7).

Comparisons by hospital type revealed no significant differences for ICC cognitive, ICC affective, compassionate care, or perspective taking, indicating that institutional setting alone does not substantially influence these competencies. However, a significant difference was detected in the standing in the patient’s shoes dimension, where nurses in private hospitals reported higher scores (t = −1.992, *p* = 0.04). This finding suggests that certain organizational environments, such as private hospitals, may provide conditions more conducive to perspective-taking behaviors, though further research is needed to clarify these contextual effects (Table 8).

The multivariate model revealed significant overall effects of age and total years of professional experience on ICC dimensions (Table 9).

The multivariate analysis indicated that demographic and professional variables jointly influenced intercultural communication competence (ICC). As presented in Table 9 (Panel A), age (Pillai’s Trace = 0.222, *p* < 0.001) and total years of professional experience (Pillai’s Trace = 0.180, *p* < 0.001) demonstrated statistically significant multivariate effects on ICC dimensions, whereas gender, hospital type, and willingly choosing the profession did not show significant overall effects.

Follow-up-adjusted univariate analyses (Panel B) revealed that both cognitive and affective ICC differed significantly by age and professional experience. Specifically, age exerted a moderate effect on cognitive ICC (*F* = 13.96, *p* < 0.001, η^2^ = 0.151) and a smaller but significant effect on affective ICC (*F* = 8.04, *p* < 0.001, η^2^ = 0.093). Similarly, total years of professional experience significantly influenced cognitive ICC (*F* = 7.96, *p* < 0.001, η^2^ = 0.092) and affective ICC (*F* = 9.38, *p* < 0.001, η^2^ = 0.107). In contrast, gender, hospital type, and willingly choosing the profession did not demonstrate statistically significant adjusted effects on either ICC dimension.

Post hoc comparisons further indicated that nurses with longer professional experience reported significantly higher ICC scores than those with fewer years of experience, suggesting a progressive increase in intercultural competence across professional tenure.

Subsequently, a MANCOVA model was conducted for the empathy subscales to examine adjusted effects of demographic and professional factors (Table 10).

The multivariate analysis of covariance demonstrated that empathy dimensions were jointly associated with age, total years of professional experience, and willingly choosing the profession (Table 10, Panel A). Significant multivariate effects were observed for age (Pillai’s Trace = 0.176, *p* < 0.001), professional experience (Pillai’s Trace = 0.129, *p* < 0.001), and willingly choosing profession (Pillai’s Trace = 0.063, *p* = 0.002), whereas gender and hospital type did not show significant overall multivariate effects.

Follow-up adjusted univariate analyses (Panel B) indicated that age significantly predicted compassionate care (*F* = 11.67, *p* < 0.001, partial η^2^ = 0.130) and perspective taking (*F* = 8.93, *p* < 0.001, partial η^2^ = 0.102) but not standing in the patient’s shoes (*F* = 0.67, *p* = 0.572). Professional experience showed significant adjusted effects on compassionate care (*F* = 6.15, *p* < 0.001, partial η^2^ = 0.073) and perspective taking (*F* = 4.81, *p* = 0.003, partial η^2^ = 0.058), while its effect on standing in the patient’s shoes was not significant (*F* = 1.83, *p* = 0.143). Importantly, willingly choosing the profession remained a significant independent predictor across all empathy dimensions after adjustment, including compassionate care (*F* = 5.73, *p* = 0.017), perspective taking (*F* = 11.51, *p* = 0.001), and standing in the patient’s shoes (*F* = 7.02, *p* = 0.009). Gender and hospital type did not yield statistically significant adjusted effects for any empathy dimension.

Bonferroni-adjusted pairwise comparisons indicated that younger nurses generally reported higher empathy. Specifically, nurses aged 20–29 scored significantly higher than those aged 40–49 and ≥50 on compassionate care and perspective taking, and higher than those aged 30–39 on perspective taking. For standing in the patient’s shoes, a significant difference was only observed between the 20–29 and 40–49 groups.

## 4. Discussion

The present study investigated nurses’ levels of intercultural communication competence (ICC) and empathy, the associations between these two constructs, and differences across key demographic and professional variables. The findings revealed that both ICC and empathy were above average, suggesting that the nursing workforce in this study generally perceives itself as competent in communicating with culturally diverse patients and in responding empathetically to their needs. Importantly, the ICC cognitive dimension scored higher than the affective dimension, while compassionate care was the strongest aspect of empathy. These results provide a nuanced understanding of how knowledge-based cultural awareness and affective relational capacities manifest in clinical settings.

The finding that nurses demonstrated relatively stronger cognitive than affective ICC resonates with earlier theoretical discussions in intercultural communication research [8,9]. Previous studies emphasize that professional education and in-service training tend to prioritize knowledge of cultural norms, beliefs, and practices rather than the emotional and relational challenges inherent in intercultural encounters [1,3]. In line with this, Spencer-Oatey and Franklin highlighted that intercultural competence requires both cognitive understanding and emotional engagement, yet formal education often privileges the former [2]. Recent empirical studies among healthcare professionals further support this pattern. Türedi et al. reported that healthcare workers exhibited higher cognitive awareness than affective sensitivity in intercultural communication, underscoring the need for affect-focused training components [21]. Similarly, Berktas and Tanriverdi found that while nurses showed moderate-to-high intercultural tolerance, affective engagement remained the most fragile component in daily practice [22]. For nursing, these findings collectively suggest the need for more experiential learning strategies -such as role plays, reflective practice, and cultural immersion- that enhance not only awareness but also affective responsiveness [10,12].

Empathy levels in the present study were also above average, with compassionate care emerging as the strongest dimension. This is consistent with classical and contemporary conceptualizations of empathy, which highlight the prominence of affective and compassionate elements in helping professions [16,18]. Empirical studies in nursing similarly emphasize that empathy is most visibly expressed through compassionate behaviors that directly influence care quality and patient satisfaction. Elliott et al. described empathy as a multidimensional construct encompassing emotional, cognitive, and behavioral processes, a framework that aligns closely with the present findings [14]. Although perspective taking and standing in the patient’s shoes scored slightly lower, they still reflected substantial levels of cognitive empathy. Comparable patterns have been observed in recent intervention studies. For example, Şahin and Ayaz-Alkaya demonstrated that cultural sensitivity education significantly increased compassion scores, while gains in perspective taking were more modest, suggesting that deeper cognitive-empathic shifts may require longer or more intensive interventions [23]. Similarly, Shin et al. reported that simulation-based training improved empathy toward foreign patients, particularly in the affective-compassionate domain, while perspective-taking improvements were smaller in magnitude [24]. The relatively lower scores in these domains may reflect workload pressures and time constraints in clinical practice, which limit nurses’ capacity to fully adopt patients’ perspectives.

One of the most salient findings of this study was the moderate to strong correlations between ICC and empathy dimensions. The total ICC score was moderately correlated with total empathy, and affective ICC showed meaningful associations with compassionate care and perspective taking. These findings support the notion that intercultural sensitivity and empathy are interdependent competencies rather than isolated skills. Philosophical and psychological perspectives describe empathy as requiring both cultural awareness and emotional resonance [14,15]. Recent quantitative evidence strengthens this interpretation. Aydin Er et al. demonstrated that compassion plays a mediating role between nurses’ ethnocultural empathy and cultural sensitivity, indicating that affective ICC functions as a key mechanism linking cultural competence to empathic care [25]. Likewise, Türker and Alpar found that transcultural effectiveness and health communication competencies jointly predicted empathic nursing behaviors in primary care settings in Türkiye [26]. Together, these findings empirically reinforce the mutual reinforcement of ICC and empathy observed in the present study.

Demographic and professional analyses revealed several noteworthy patterns. Male nurses scored higher on both cognitive and affective ICC dimensions, diverging from much of the literature that often reports higher empathic tendencies among women [21]. This discrepancy may be influenced by contextual and cultural factors, particularly within the Turkish nursing workforce, where male nurses may experience greater pressure to demonstrate adaptability and intercultural competence in a predominantly female profession. Notably, empathy did not differ significantly by gender, supporting arguments that empathic capacity in professional nursing is shaped more by role socialization and professional norms than by biological sex [22].

Age-related differences were also prominent. Younger nurses (20–29 years) scored highest in both ICC and empathy, while the oldest group scored lowest. These findings align with theories suggesting that younger generations are more likely to embrace multicultural identities due to globalization and educational exposure [5]. However, professional experience also emerged as a positive factor, as nurses with ≥28 years of experience demonstrated higher ICC and empathy, particularly in compassionate care and perspective taking. This dual pattern mirrors recent findings by Türedi et al. and Berktas and Tanriverdi, who reported that both generational openness and cumulative clinical experience contribute to intercultural competence, albeit through different mechanisms [21,22]. Younger nurses may bring openness and adaptability, whereas experienced nurses develop empathy and cultural sensitivity through repeated exposure to diverse patient populations over time [10,12].

The most pronounced differences were observed between nurses who willingly chose the profession and those who did not. Nurses who entered nursing by choice demonstrated significantly higher ICC and empathy across all dimensions. This finding aligns with theories emphasizing the role of motivation and identity in shaping intercultural adaptability and empathic engagement [3,4]. Recent studies corroborate this association, showing that intrinsic motivation and professional commitment are strong predictors of cultural sensitivity, compassion, and empathic communication in nursing practice [25,26]. These results underscore intrinsic motivation as a critical lever for fostering culturally responsive care.

Finally, hospital type showed minimal differences, with the exception that nurses working in private hospitals scored higher on the “standing in the patient’s shoes” dimension. This pattern may be associated with organizational cultures that more explicitly prioritize patient experience, individualized care, and service quality indicators, which are often central to the operational models of private healthcare institutions. In such settings, performance evaluation systems, patient satisfaction monitoring, and communication standards may place stronger emphasis on relational competencies, including perspective taking. Moreover, relatively structured care pathways and managerial accountability mechanisms in private hospitals may create clearer expectations regarding empathic engagement and patient-centered interaction, thereby reinforcing role-reversal capacities in daily practice. Consistent with this interpretation, prior research suggests that organizational climate and institutional norms can shape empathic communication behaviors more strongly than cognitive cultural awareness alone [26]. Nevertheless, effective communication and empathy remain essential across all healthcare settings, and structural constraints often limit their full enactment regardless of institution type [22].

Taken together, the findings of this study provide robust empirical evidence that ICC and empathy are interrelated and generally above average among nurses yet unevenly distributed across demographic and professional characteristics. While cognitive cultural awareness appears relatively strong, affective ICC and certain empathy subdimensions require further development. By integrating recent national and international evidence [23,24,25,26], this study extends the literature by situating its findings within a broader empirical context and highlighting both individual-level (motivation, age, experience) and organizational-level factors that shape culturally responsive nursing care. These insights offer practical guidance for educators, managers, and policymakers aiming to strengthen patient-centered, culturally competent healthcare systems.

### Limitations

This study employed a cross-sectional, self-report design, which does not allow causal inference and reflects perceived rather than directly observed competencies. The sample was drawn from a single national context, which may limit transferability to other healthcare systems. Although validated instruments were used, standardized scales may not fully capture the complexity of intercultural and empathic behaviors in real clinical interactions. Future research incorporating longitudinal, multi-site, or mixed-method designs could further extend these findings.

## 5. Conclusions

Nurses in this sample reported above-average intercultural communication competence and empathy, with stronger cognitive than affective ICC and compassionate care as the most pronounced empathic facet. ICC and empathy were moderately to strongly interrelated, and varied by age, experience, and professional motivation, but not meaningfully by hospital type. These findings underscore the value of integrating ICC and empathy development in nursing education and in-service training, prioritizing experiential, reflective, and scenario-based methods that strengthen affective ICC and perspective taking. Future work should employ longitudinal and mixed-methods designs, include multi-site samples, and link competency gains to patient outcomes to establish causality and practical impact.

## Figures and Tables

**Table 1:** Demographic characteristics of participants (n = 300).

Variable	Category	n	%
Gender	Male	93	31.0
	Female	207	69.0
Age	20–29	78	26.0
	30–39	95	31.7
	40–49	72	24.0
	≥50	55	18.3
Marital status	Single	138	46.0
	Married	162	54.0
Total years of experience	1–9	121	40.3
	10–18	68	22.7
	19–27	61	20.3
	≥28	50	16.7
Chose profession willingly	Yes	254	84.7
	No	46	15.3
Works with enjoyment	Yes	262	87.3
	No	38	12.7
Hospital type	State	218	72.7
	Private	82	27.3
Work shift	Permanent day	100	33.3
	Permanent night	20	6.7
	Rotating (day–night)	180	60.0

**Table 2:** Descriptive statistics for intercultural communication competence (ICC) and empathy (n = 300).

Scale/Dimension	Mean	SD
Intercultural Communication Competence (ICC)		
Cognitive	4.71	1.42
Affective	4.35	1.34
Total ICC	4.11	0.80
Empathy		
Compassionate care	4.60	1.10
Perspective taking	4.44	0.99
Standing in the patient’s shoes	4.45	1.42
Total empathy	4.50	0.90

**Table 3:** Correlations between ICC and empathy.

	ICC—Cognitive	ICC—Affective	ICC—Total	Compassionate Care	Perspective Taking	Standing in the Patient’s Shoes	Empathy—Total
ICC—Cognitive	1						
ICC—Affective	0.332 *	1					
ICC—Total	0.767 *	0.860 *	1				
Compassionate care	0.412 *	0.455 *	0.533 *	1			
Perspective taking	0.420 *	0.493 *	0.563 *	0.641 *	1		
Standing in the patient’s shoes	0.307 *	0.156 *	0.272 *	0.296 *	0.347 *	1	
Empathy—Total	0.478 *	0.512 *	0.607 *	0.875 *	0.911 *	0.504 *	1

Note: * *p* < 0.05.

**Table 4:** Group differences by gender (independent-samples *t*-tests).

Outcome	Gender	n	Mean	SD	df	t	*p*
ICC—Cognitive	Male	93	5.01	1.47	298	2.497	0.013 *
	Female	207	4.57	1.37			
ICC—Affective	Male	93	4.69	1.26	298	2.497	0.002 *
	Female	207	4.19	1.34			
Compassionate care	Male	93	4.67	1.12	298	0.845	0.399
	Female	207	4.56	1.08			
Perspective taking	Male	93	4.58	0.93	298	1.787	0.075
	Female	207	4.36	1.00			
Standing in the patient’s shoes	Male	93	4.56	1.51	298	0.993	0.321
	Female	207	4.39	1.37			

Note: * *p* < 0.05.

**Table 5:** Group differences by age (one-way ANOVA; Scheffé post hoc).

Outcome	Age Group	n	Mean	SD	*F*	*p*	Significant Scheffé Contrasts
ICC—Cognitive	20–29	78	5.376	1.259	13.643	0.000 *	20–29 > 30–39, 50+; 30–39 > 50+; 40–49 > 50+
	30–39	95	4.583	1.369			
	40–49	72	4.787	1.152			
	≥50	55	3.897	1.580			
ICC—Affective	20–29	78	4.926	1.267	8.209	0.000 *	20–29 > 30–39, 40–49, 50+
	30–39	95	4.305	1.224			
	40–49	72	4.132	1.324			
	≥50	55	3.891	1.397			
Compassionate care	20–29	78	4.940	0.895	7.140	0.000 *	20–29 > 50+; 30–39 > 50+
	30–39	95	4.636	0.974			
	40–49	72	4.581	1.125			
	≥50	55	4.075	1.326			
Perspective taking	20–29	78	4.866	0.723	13.153	0.000 *	20–29 > 40–49, 50+; 30–39 > 50+
	30–39	95	4.516	0.901			
	40–49	72	4.310	0.954			
	≥50	55	3.861	1.192			
Standing in the patient’s shoes	20–29	78	4.750	1.328	2.093	0.101	— (ns)
	30–39	95	4.474	1.309			
	40–49	72	4.222	1.448			
	≥50	55	4.273	1.638			

Notes: * *p* < 0.05; ns = not significant.

**Table 6:** Group differences by total years of experience (one-way ANOVA; Scheffé post hoc).

Outcome	Experience	n	Mean	SD	*F*	*p*	Significant Scheffé Contrasts
ICC—Cognitive	1–9	121	4.46	1.61	3.464	0.017 *	1–9 < ≥28
	10–18	68	4.67	1.24			
	19–27	61	4.83	1.23			
	≥28	50	5.20	1.21			
ICC—Affective	1–9	121	4.05	1.39	8.047	0.000 *	1–9 < ≥28; 10–18 < ≥28
	10–18	68	4.19	1.37			
	19–27	61	4.50	1.20			
	≥28	50	5.08	1.00			
Compassionate care	1–9	121	4.30	1.22	8.660	0.000 *	1–9 < 19–27, ≥28; 10–18 < ≥28
	10–18	68	4.53	0.92			
	19–27	61	4.77	0.99			
	≥28	50	5.17	0.84			
Perspective taking	1–9	121	4.24	1.02	7.786	0.000 *	1–9 < ≥28; 10–18 < ≥28; 19–27 < ≥28
	10–18	68	4.42	0.90			
	19–27	61	4.34	0.97			
	≥28	50	5.01	0.82			
Standing in the patient’s shoes	1–9	121	4.41	1.48	3.594	0.014 *	Omnibus significant; no Scheffé pairwise significant
	10–18	68	4.33	1.28			
	19–27	61	4.18	1.47			
	≥28	50	5.01	1.25			

Note: * *p* < 0.05.

**Table 7:** Group differences by willingly choosing the profession (independent-samples *t*-tests).

Outcome	Willingly Chose Profession	n	Mean	SD	df	t	*p*
ICC—Cognitive	Yes	254	4.86	1.33	298	4.482	0.000 *
	No	46	3.87	1.55			
ICC—Affective	Yes	254	4.50	1.26	298	5.053	0.000 *
	No	46	3.46	1.40			
Compassionate care	Yes	254	4.74	0.96	298	5.677	0.000 *
	No	46	3.79	1.38			
Perspective taking	Yes	254	4.58	0.89	298	6.319	0.000 *
	No	46	3.64	1.12			
Standing in the patient’s shoes	Yes	254	4.57	1.37	298	3.639	0.000 *
	No	46	3.76	1.48			

Note: * *p* < 0.05.

**Table 8:** Group differences by hospital type (independent-samples *t*-tests).

Outcome	Hospital Type	n	Mean	SD	df	t	*p*
ICC—Cognitive	State	218	4.71	1.43	298	−0.024	0.980
	Private	82	4.71	1.37			
ICC—Affective	State	218	4.33	1.34	298	−0.350	0.720
	Private	82	4.39	1.33			
Compassionate care	State	218	4.58	1.11	298	−0.424	0.670
	Private	82	4.64	1.04			
Perspective taking	State	218	4.40	1.01	298	−1.007	0.310
	Private	82	4.53	0.91			
Standing in the patient’s shoes	State	218	4.34	1.45	298	−1.992	0.040 *
	Private	82	4.71	1.30			

Note: * *p* < 0.05.

**Table 9:** Multivariate and adjusted univariate effects of demographic and professional variables on intercultural communication competence (ICC).

Panel A. Multivariate Effects (MANCOVA)						
Factor	Pillai’s Trace	*F*	df	*p*	Partial η^2^	
Gender	0.012	1.44	2, 234	0.239	0.012	
**Age**	**0.222**	**9.76**	6, 470	**<0.001**	0.111	
**Total years of experience**	**0.180**	**7.73**	6, 470	**<0.001**	0.090	
Willingly chose profession	0.017	1.97	2, 234	0.142	0.017	
Hospital type	0.002	0.18	2, 234	0.834	0.002	
**Panel B. Adjusted Univariate Effects**						
**Factor**	**Cognitive ICC *F***	***p***	**η^2^**	**Affective ICC *F***	***p***	**η^2^**
Gender	1.58	0.210	0.007	1.37	0.243	0.006
**Age**	**13.96**	**<0.001**	0.151	**8.04**	**<0.001**	0.093
**Total experience**	**7.96**	**<0.001**	0.092	**9.38**	**<0.001**	0.107
Willingly chose profession	0.55	0.459	0.002	3.46	0.064	0.015
Hospital type	0.00	0.977	0.000	0.36	0.547	0.002

Notes: Pillai’s Trace is reported as the most robust multivariate statistic. Significant values are shown in bold. ICC = Intercultural Communication Competence.

**Table 10:** Multivariate and adjusted univariate effects of demographic and professional variables on empathy dimensions.

Panel A. Multivariate Effects (MANCOVA)									
Factor	Pillai’s Trace	*F*		df		*p*		Partial η^2^	
Gender	0.021	1.700		3, 233		0.168		0.021	
**Age**	0.176	4.869		9, 705		<0.001		0.059	
**Total years of experience**	0.129	3.528		9, 705		<0.001		0.043	
**Willingly chose profession**	0.063	5.190		3, 233		0.002		0.063	
Hospital type	0.005	0.390		3, 233		0.760		0.005	
**Panel B. Adjusted Univariate Effects**									
**Factor**	**Compassionate Care *F***	***p***	**η^2^**	**Perspective Taking *F***	***p***	**η^2^**	**Standing in Patient’s Shoes *F***	***p***	**η^2^**
Gender	2.998	0.085	0.013	0.093	0.761	0.000	2.526	0.113	0.011
**Age**	**11.672**	**<0.001**	0.130	**8.928**	**<0.001**	0.102	0.669	0.572	0.008
**Total experience**	**6.146**	**<0.001**	0.073	**4.806**	**0.003**	0.058	1.826	0.143	0.023
**Willingly chose profession**	**5.734**	**0.017**	0.024	**11.505**	**0.001**	0.047	**7.016**	**0.009**	0.029
Hospital type	1.014	0.315	0.004	0.155	0.694	0.001	0.038	0.846	0.000

Notes: Pillai’s Trace was reported as the most robust multivariate statistic. Significant effects are shown in bold. Bonferroni adjustment was applied in pairwise comparisons.

## Data Availability

The data presented in this study are available on request from the corresponding author. The data are not publicly available due to ethical and privacy restrictions related to participant confidentiality.

## Abbreviations

The following abbreviations are used in this manuscript:

ICC	Intercultural Communication Competence
JSE	Jefferson Scale of Empathy
EFA	Exploratory Factor Analysis
CFA	Confirmatory Factor Analysis
